# Supplementary material for: Adaptive laboratory evolution enhances methanol tolerance and conversion in engineered Corynebacterium glutamicum
Source: Commun Biol. 2020 May 7;3:217. doi: 10.1038/s42003-020-0954-9 (PMC7205612; doi:10.1038/s42003-020-0954-9)
Supplement: Supplementary file 1 — Supplementary Information [file 42003_2020_954_MOESM1_ESM.pdf]

Supplementary information for

Adaptive laboratory evolution enhances methanol tolerance and conversion in engineered *Corynebacterium glutamicum*

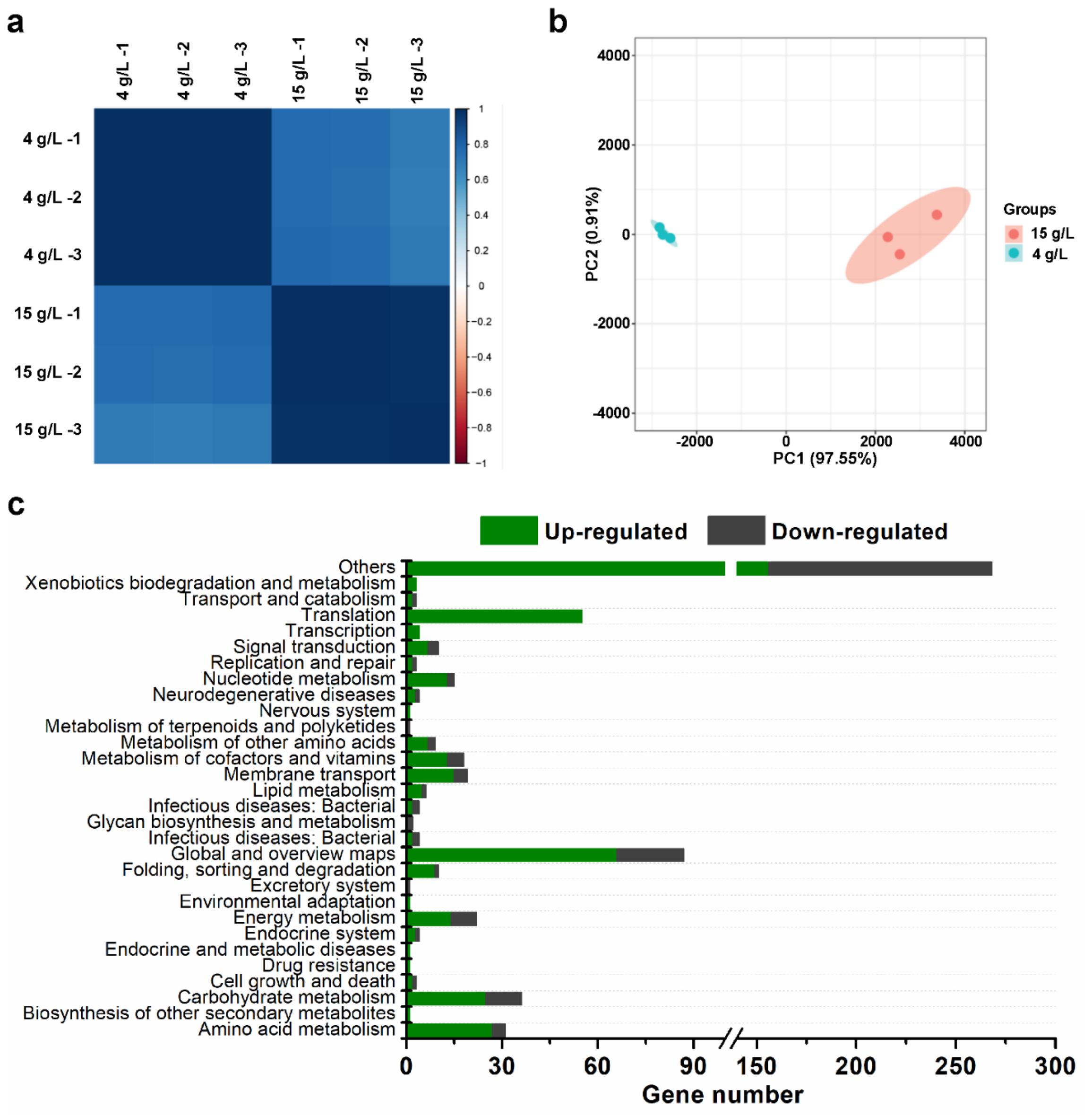

Supplementary Fig. 1 Evaluation of the accuracy and repeatability of transcriptome analysis and classification of differentially expressed genes. **a**, Pearson's correlation coefficient test. **b**, Principle component analysis (PCA). **c**, Classification of differentially expressed genes according to KEGG_B_class annotation.

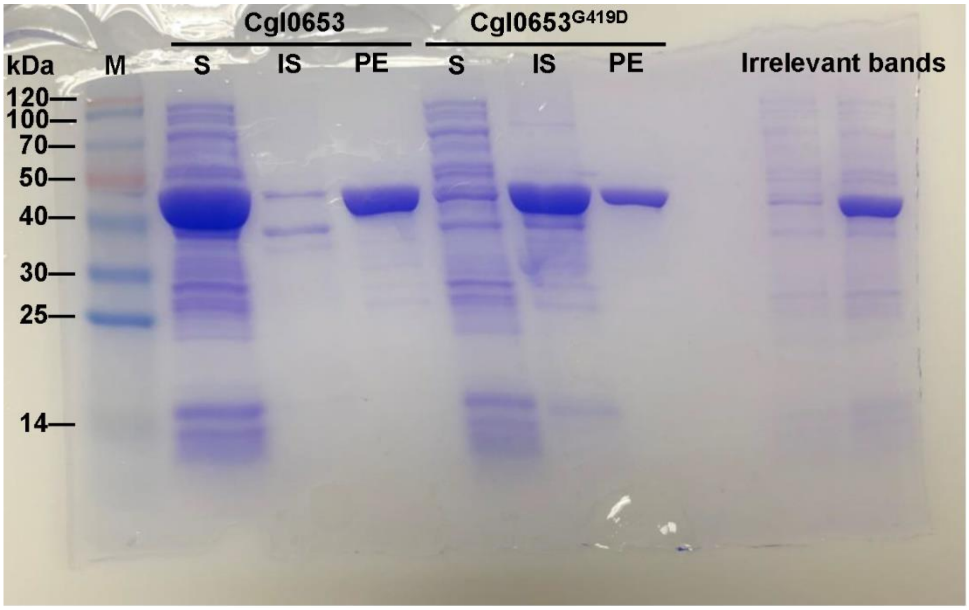

Supplementary Fig. 2 The uncropped gel image of that shown in Fig. 5b. S, Soluble supernatant of cell extract; IS, insoluble sediment; PE, purified enzyme.

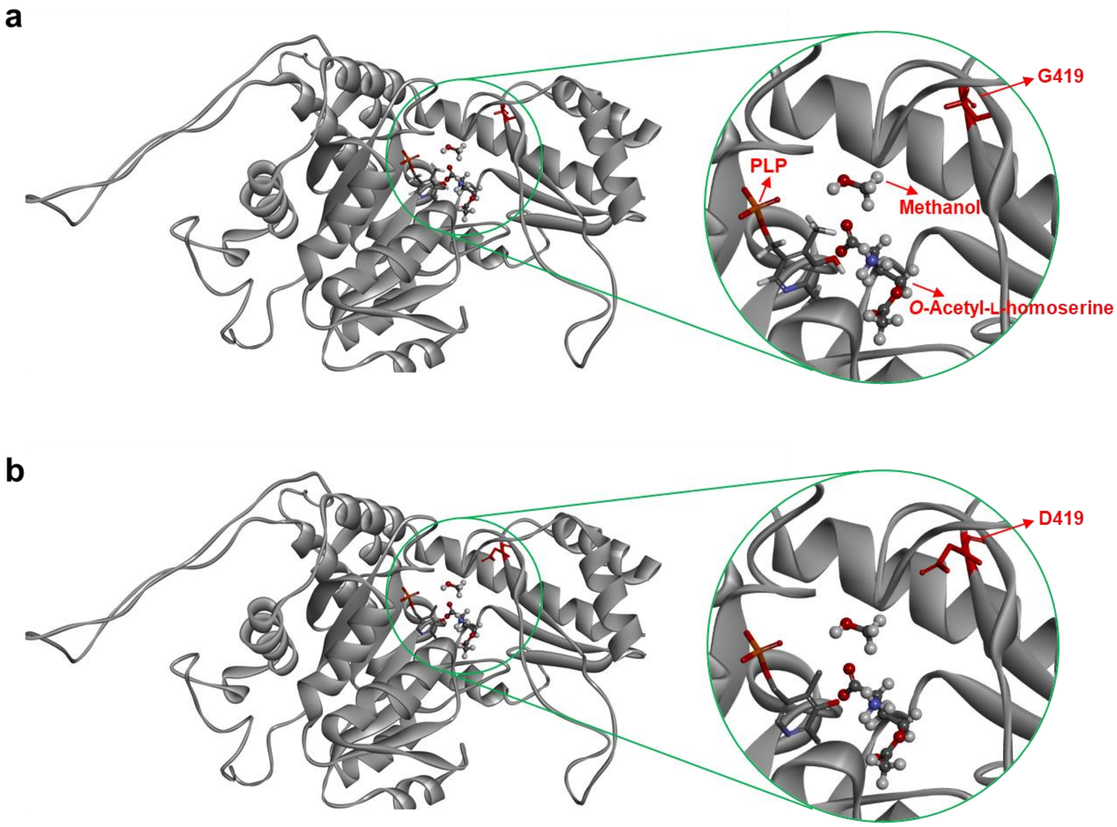

Supplementary Fig. 3 Mapping G419D mutation in Cgl0653 (MetY). The model structure of the wild-type Cgl0653 was constructed with the crystal structure of *O*-acetyl-L-homoserine sulfhydrylase from *Mycobacterium marinum* ATCC BAA-535 (PDB ID: 4KAM) as a template (54% sequence identity with Cgl0653) using Discovery Studio 4.1 software (Biovia). Flexible dockings of pyridoxal-5-phosphate (PLP), methanol and *O*-acetyl-L-homoserine into the active site were performed with Discovery Studio 4.1 software (Biovia). **a**, Wild-type Cgl0653. **b**, Cgl0653^{G419D}.

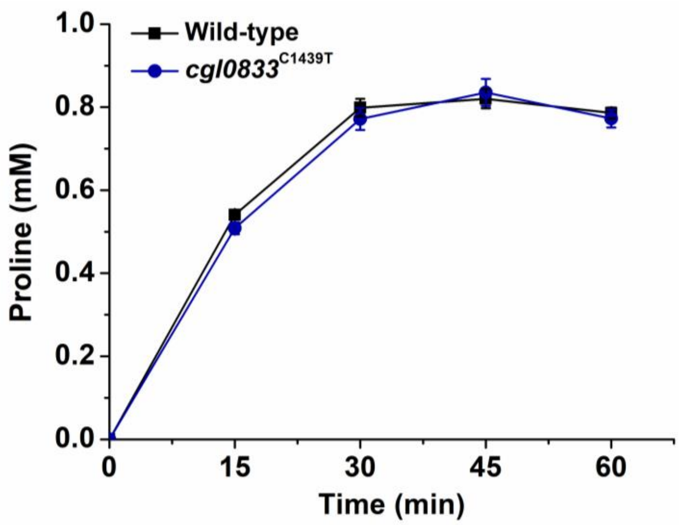

Supplementary Fig. 4 Proline transport by *C. glutamicum* ATCC 13032 wild-type strain and *cgl0833*^{C1439T} mutant strain. Pregrown cells were harvested and washed twice with ice-cold CGXII medium. The peptide uptake and amino acid excretion were then initiated by resuspending the cells in prewarmed CGXII medium (30°C) containing 2 mM Phe-Pro. The resulting cell density (OD_{600nm}) was 10.0. The cells were incubated at 30°C and with shaking at 220 rpm. Samples were taken every 15 min, and extracellular amino acids were quantified using HPLC. Error bars indicate standard deviations from three parallel experiments.

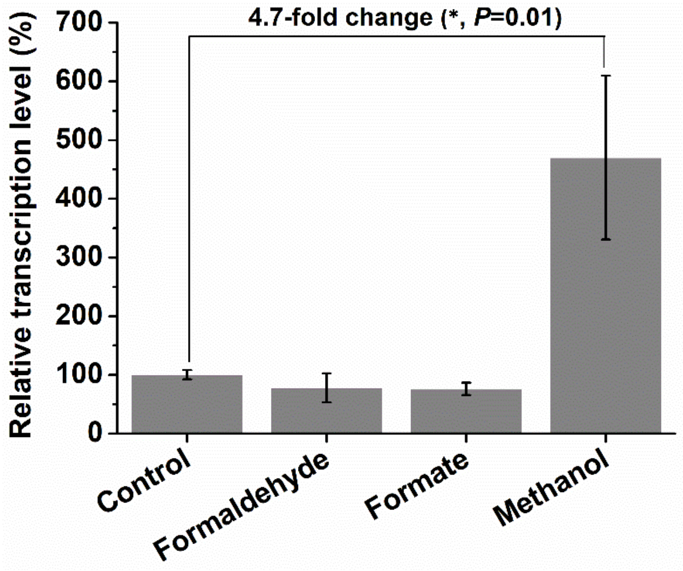

Supplementary Fig. 5 Relative transcription level of *cgl0833* in *C. glutamicum* ATCC 13032 under treatment with methanol (5 g/L), formaldehyde (15 mg/L), or formate (5 g/L). Error bars indicate standard deviations from three parallel experiments. *P<0.05, one-way ANOVA, N=3.

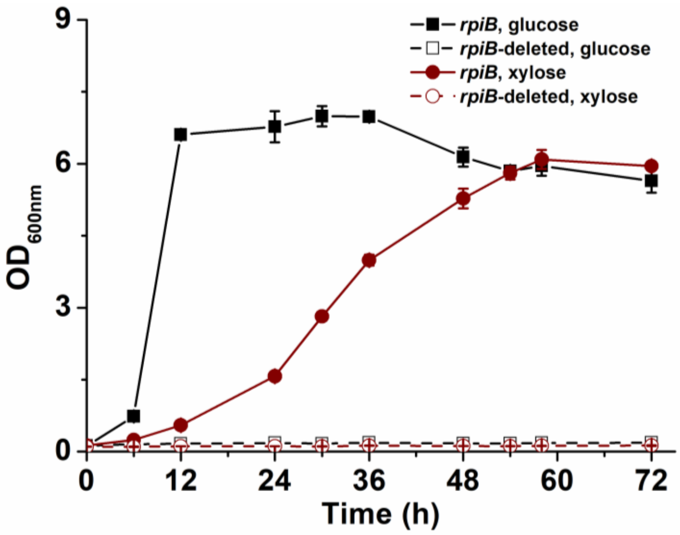

Supplementary Fig. 6 Effects of *rpiB* deletion on cell growth on xylose and glucose. *C. glutamicum* strains were cultivated using CGXII minimal medium supplemented with 4 g/L glucose or 4 g/L xylose as the carbon source. Error bars indicate standard deviations from three parallel experiments.

Supplementary Data 1 Gene transcript level changes between *C. glutamicum* strain MX-14 cultivated with 15 g/L vs. 4 g/L methanol

Supplementary Data 1 is provided separately as an excel document.

Supplementary Table 1 *In silico* analysis of *rpiB* deletion on cell growth with different carbon sources[a]

**Carbon source**[b]	**Cell growth (h^{-1})**
Xylose	0
Glucose	0
Methanol and xylose	0.081
Methanol and glucose	0.116

[a]The reaction catalyzed by RpiB (R5P <=> Ru5P) was deleted from the genome-scale metabolic model of *C. glutamicum* ATCC13032, *i*CW773[1].

[b]Uptake rate of each carbon source was set as 1 mmol/gCDW·h.

Supplementary Table 2 Strains and plasmids used in this study

Strain or plasmid	Description[a]	Reference or source
**Strain**		
E. coli		
DH5α	General cloning host	TaKaRa
BL21 (DE3)	Gene overexpression host	Novagen
BL21 (pET-28a-*metX*)	Derivative of BL21 (DE3) harboring pET-28a-*metX* for heterogeneous expression of *metX* from *Leptospira meyeri* fused with a N-terminal His·Tag	Lab stock
BL21 (pET-21a-*cgl0653*)	Derivative of BL21 (DE3) harboring pET-21a-*cgl0653*	This study
BL21 (pET-21a-*cgl0653*^{G1256A})	Derivative of BL21 (DE3) harboring pET-21a-*cgl0653*^{G1256A}	This study
C. glutamicum		
ATCC 13032	Wild-type strain	Lab stock
MX-4	Mutant of strain MX-3 that grows fast using ribose and xylose as carbon sources	[2]
MX-10	Derivative of strain MX-4 harboring pEC-XK99E-mdh_{Bs2334}-*hps*-phi_{Bm}	[2]
MX-11	Mutant of strain MX-10 that grows fast using methanol and xylose as carbon sources	[2]
MX-12	Mutant of strain MX-11 that grows fast using xylose and high concentrations of methanol as carbon sources	This study
MX-13	Mutant of strain MX-11 that grows fast using xylose and high concentrations of methanol as carbon sources	This study
MX-14	Mutant of strain MX-11 that grows fast using xylose and high concentrations of methanol as carbon sources	This study
MX-10-*cgl0653*^{G1256A}	Derivative of strain MX-10 harboring *cgl0653*^{G1256A} mutation	This study
MX-10-*cgl0754*^{C582A}	Derivative of strain MX-10 harboring *cgl0754*^{C582A} mutation	This study
MX-10-*cgl1367*^{C584T}	Derivative of strain MX-10 harboring *cgl1367*^{C584T} mutation	This study
MX-10-*cgl1520*^{A574G}	Derivative of strain MX-10 harboring *cgl1520*^{A574G} mutation	This study
MX-10-*cgl2998*^{G104T}	Derivative of strain MX-10 harboring *cgl2998*^{G104T} mutation	This study
MX-10-*cgl2365*^{C542G}	Derivative of strain MX-10 harboring *cgl2365*^{C542G} mutation	This study

MX-10-*cgl2857*^{G183A}	Derivative of strain MX-10 harboring *cgl2857*^{G183A} mutation	This study
MX-10-*cgl0833*^{C1439T}	Derivative of strain MX-10 harboring *cgl0833*^{C1439T} mutation	This study
13032-*cgl0653*^{G1256A}	Derivative of strain ATCC 13032 harboring *cgl0653*^{G1256A} mutation	This study
13032-*cgl0754*^{C582A}	Derivative of strain ATCC 13032 harboring *cgl0754*^{C582A} mutation	This study
13032-*cgl1367*^{C584T}	Derivative of strain ATCC 13032 harboring *cgl1367*^{C584T} mutation	This study
13032-*cgl1520*^{A574G}	Derivative of strain ATCC 13032 harboring *cgl1520*^{A574G} mutation	This study
13032-*cgl2998*^{G104T}	Derivative of strain ATCC 13032 harboring *cgl2998*^{G104T} mutation	This study
13032-*cgl2365*^{C542G}	Derivative of strain ATCC 13032 harboring *cgl2365*^{C542G} mutation	This study
13032-*cgl2857*^{G183A}	Derivative of strain ATCC 13032 harboring *cgl2857*^{G183A} mutation	This study
13032-*cgl0833*^{C1439T}	Derivative of strain ATCC 13032 harboring *cgl0833*^{C1439T} mutation	This study
13032-*cgl0833-gfp*	Derivative of strain ATCC 13032 with *gfp* fused to *cgl0833*	This study
13032-*cgl0833*^{C1439T}-*gfp*	Derivative of strain 13032-*cgl0833*^{C1439T} with *gfp* fused to *cgl0833*^{C1439T}	This study
13032 (pdCas9-gRNA-*cgl0653*)	Derivative of strain ATCC 13032 harboring pdCas9-gRNA-*cgl0653*	This study
13032 (pdCas9-gRNA-*cgl0833*)	Derivative of strain ATCC 13032 harboring pdCas9-gRNA-*cgl0833*	This study
13032 (pEC-XK99E-*cgl0653*)	Derivative of strain ATCC 13032 harboring pEC-XK99E-*cgl0653*	This study
13032 (pEC-XK99E-*cgl0653*^{G1256A})	Derivative of strain ATCC 13032 harboring pEC-XK99E-*cgl0653*^{G1256A}	This study
13032 (pEC-XK99E-*cgl0833*)	Derivative of strain ATCC 13032 harboring pEC-XK99E-*cgl0833*	This study
13032 (pEC-XK99E-*cgl0833*^{C1439T})	Derivative of strain ATCC 13032 harboring pEC-XK99E-*cgl0833*^{C1439T}	This study
13032Δ*cgl0653*	Derivative of strain ATCC 13032 with *cgl0653* knocked out	This study
13032Δ*cgl0833*	Derivative of strain ATCC 13032 with *cgl0833* knocked out	This study
**Plasmid**		
pET-21a(+)	Overexpression vector, C-terminal His·Tag, Amp^{R}	Novagen
pK18*mobsacB*	Suicide vector for genome editing in *C. glutamicum*, *mob*, *sacB*, Km^{R}	[3]

pEC-XK99E	Expression vector, IPTG-inducible promoter P_{trc}, Km^{R}	4
pdCas9	pXMJ19 carrying dCas9 cassette driven by IPTG-inducible promoter P_{tac} for gene knock-down, Cm^{R}	5
pnCas9(D10A)-AID-gRNA-*ccdB*^{TS}	All-in-one plasmid for base editing in *C. glutamicum*	6
pEC-XK99E-*mdh*$_{Bs2334}$-*hps-phi*$_{Bm}$	pEC-XK99E harboring *mdh* gene from *Bacillus stearothermophilus* DSM 2334, under the control of P_{trc}, and *hps* and *phi* genes from *B. methanolicus* MGA3, under the control of constitutive promoter P_{P5}	2
pK18-*cgl0653*^{G1256A}	pK18*mobsacB* derivative for introducing *cgl0653*^{G1256A} mutation	This study
pK18-*cgl0754*^{C582A}	pK18*mobsacB* derivative for introducing *cgl0754*^{C582A} mutation	This study
pK18-*cgl1367*^{C584T}	pK18*mobsacB* derivative for introducing *cgl1367*^{C584T} mutation	This study
pK18-*cgl1520*^{A574G}	pK18*mobsacB* derivative for introducing *cgl1520*^{A574G} mutation	This study
pK18-*cgl2998*^{G104T}	pK18*mobsacB* derivative for introducing *cgl2998*^{G104T} mutation	This study
pK18-*cgl2365*^{C542G}	pK18*mobsacB* derivative for introducing *cgl2365*^{C542G} mutation	This study
pK18-*cgl2857*^{G183A}	pK18*mobsacB* derivative for introducing *cgl2857*^{G183A} mutation	This study
pK18-*cgl0833*^{C1439T}	pK18*mobsacB* derivative for introducing *cgl0833*^{C1439T} mutation	This study
pdCas9-gRNA-*ccdB*	All-in-one plasmid for CRISPRi, pdCas9 derivative with gRNA-*ccdB* cassette from pgRNA-*ccdB*	This study
pdCas9-gRNA-*cgl0653*	pdCas9-gRNA-*ccdB* derivative harboring gRNA targeting *cgl0653* for knock-down	This study
pdCas9-gRNA-*cgl0833*	pdCas9-gRNA-*ccdB* derivative harboring gRNA targeting *cgl0833* for knock-down	This study
pEC-XK99E-*cgl0653*	pEC-XK99E derivative harboring wild-type *cgl0653* for overexpression	This study
pEC-XK99E-*cgl0653*^{G1256A}	pEC-XK99E derivative harboring *cgl0653*^{G1256A} mutant for overexpression	This study
pEC-XK99E-*cgl0833*	pEC-XK99E derivative harboring wild-type *cgl0833* for overexpression	This study
pEC-XK99E-*cgl0833*^{C1439T}	pEC-XK99E derivative harboring *cgl0833*^{C1439T} mutant for overexpression	This study
pK18-*cgl0833-gfp*	pK18*mobsacB* derivative harboring homologous arms and *gfp* for fusing *gfp* with *cgl0833*	This study

pK18-*cgl0833*^{C1439T}-*gfp*	pK18*mobsacB* derivative harboring homologous arms and *gfp* for fusing *gfp* with *cgl0833*^{C1439T}	This study
pET-21a-*cgl0653*	pET-21a(+) derivative harboring *cgl0653* fused with a C-terminal His·Tag	This study
pET-21a-*cgl0653*^{G1256A}	pET-21a(+) derivative harboring *cgl0653*^{G1256A} fused with a C-terminal His·Tag	This study
pK18-Δ*cgl0653*	pK18*mobsacB* derivative harboring homologous arms for *cgl0653* knock-out	This study
pK18-Δ*cgl0833*	pK18*mobsacB* derivative harboring homologous arms for *cgl0833* knock-out	This study

[a]Amp^{R}, Km^{R} and Cm^{R} represent resistance to ampicillin, kanamycin, and chloramphenicol, respectively.

Supplementary Table 3 Primers used in this study

**Primer**	**Sequence (5'-3')**	**Relevance**
cgl0653^{G1256A}-F	TATGACATGATTACGAATTCCACGCTGTAGCGTTCTCCTC	pK18-*cgl0653*^{G1256A}
cgl0653^{G1256A}-R	CGACGGCCAGTGCCAAGCTTGCTGTACTAGCTTGTCTGCTTGGTAG	
cgl0754^{C582A}-F	TATGACATGATTACGAATTCTTGATGACTTAAATGCGCCCGGC	pK18-*cgl0754*^{C582A}
cgl0754^{C582A}-R	CGACGGCCAGTGCCAAGCTTGCGGTGTGCGCAATTCGTG	
cgl1367^{C584T}-F	TATGACATGATTACGAATTCCCGGTATTCCATCTTCAGGTTGTACTG	pK18-*cgl1367*^{C584T}
cgl1367^{C584T}-R	CGACGGCCAGTGCCAAGCTTGACCAGCAATGGATCGGCAC	
cgl1520^{A574G}-F	TATGACATGATTACGAATTCCACCTTGTCCGCCCACAGC	pK18-*cgl1520*^{A574G}
cgl1520^{A574G}-R	CGACGGCCAGTGCCAAGCTTACCATGATCCGCTAACAACCGC	
cgl2998^{G104T}-F	TATGACATGATTACGAATTCGTTGACCTTGTTCGTGGCTATGC	pK18-*cgl2998*^{G104T}
cgl2998^{G104T}-R	CGACGGCCAGTGCCAAGCTTGCGGAGCATCCGAGAAGTTC	
cgl2365^{C542G}-F	TATGACATGATTACGAATTCCAGCTGGGGCAGCGTTGAG	pK18-*cgl2365*^{C542G}
cgl2365^{C542G}-R	CGACGGCCAGTGCCAAGCTTACGGACGGTTGGAACATTTGCG	
cgl2857^{G183A}-F	TATGACATGATTACGAATTCTGCCGAGCGTTTTCATCCAACTG	pK18-*cgl2857*^{G183A}
cgl2857^{G183A}-R	CGACGGCCAGTGCCAAGCTTCGGCCAAAAACTTGGAAGGCC	
cgl0833^{C1439T}-F	TATGACATGATTACGAATTCTACTTGATCGCTCAGATGGCTGG	pK18-*cgl0833*^{C1439T}
cgl0833^{C1439T}-R	CGACGGCCAGTGCCAAGCTTAGAGGAGTGCAGCATGAGATCATC	
dCas9-F	AAACAGAATTAATTAAGCTTAAAGGAGTTG	pdCas9-gRNA-*ccdB*
dCas9-R	CCTCTAGAGTCGACCTGCAG	
CRISPRi$_{cgl0653}$-F	TTCAAAAGCCCCACTGGTCAGCAT	pdCas9-gRNA-*cgl0653*
CRISPRi$_{cgl0653}$-R	AAACATGCTGACCAGTGGGGCTTT	
CRISPRi$_{cgl0833}$-F	TTCACAGAAACAGCGTCTTGTGCA	pdCas9-gRNA-*cgl0833*
CRISPRi$_{cgl0833}$-R	AAACTGCACAAGACGCTGTTTCTG	

cgl0653-F	ACAGGCCAAAGGAGTTGAGAATGCCAAAGTACGACAATTCCA	pEC-XK99E-*cgl0653* and
cgl0653-R	CCAAGCTTGCATGCCTGCAGCTAGATTGCAGCAAAGCCGC	pEC-XK99E-*cgl0653*^{G1256A}
cgl0833-F	ACAGGCCAAAGGAGTTGAGAATGAATTCCACTATTCTCCTTGC	pEC-XK99E-*cgl0833* and
cgl0833-R	CCAAGCTTGCATGCCTGCAGTTAGTGATCAACAGCCTTTTCAAC	pEC-XK99E-*cgl0833*^{C1439T}
cgl0833-*gfp*-F1	GAGCTCGGTACCCGGGGATCCATTATGACCGTTCTGACCTTCGT	pK18-*cgl0833*-*gfp* and pK18-
cgl0833-*gfp*-R1	AGCTCCTCGCCCTTGCTCACGTGATCAACAGCCTTTTCAACA	*cgl0833*^{C1439T}-*gfp*
cgl0833-*gfp*-F2	GTGAGCAAGGGCGAGGAGC	
cgl0833-*gfp*-R2	TTACTTGTACAGCTCGTCCATGC	
cgl0833-*gfp*-F3	TGGACGAGCTGTACAAGTAAATCTAGTTTCTGAAGTTATTTAAACCG	
cgl0833-*gfp*-R3	CAGGTCGACTCTAGAGGATCCCCCACCATTCCTGGAAACTC	
cgl0653-21a-F	AAGAAGGAGATATACATATGCCAAAGTACGACAATTCCAATG	pET-21a-*cgl0653* and
cgl0653-21a-R	TGGTGGTGGTGGTGCTCGAGGATTGCAGCAAAGCCGCCT	pET-21a-*cgl0653*^{G1256A}
Δ*cgl0653*-F1	GAGCTCGGTACCCGGGGATCCGCCCATTTCATCACGAACCAT	pK18-Δ*cgl0653*
Δ*cgl0653*-R1	AATGGGTGGTGTACTTTGGCATTTGGAGGTCCT	
Δ*cgl0653*-F2	GCCAAAGTACACCACCCATTCACAGTCCGA	
Δ*cgl0653*-R2	CAGGTCGACTCTAGAGGATCCCGTTCTTTTGGGCTTTGGTG	
Δ*cgl0833*-F1	GAGCTCGGTACCCGGGGATCCGAAGTGTGTTCCATGCCCCA	pK18-Δ*cgl0833*
Δ*cgl0833*-R1	CACCGACACCCGTCTTGTGCAAGGAGAATAGTG	
Δ*cgl0833*-F2	GCACAAGACGGGTGTCGGTGTTGAAAAGGCT	
Δ*cgl0833*-R2	CAGGTCGACTCTAGAGGATCCACTCCAACACGGGAACAGGTACT	
16s-qF	ATAACTTGAGTGCTGTAGG	qPCR
16s-qR	TTGGTGTTCCTCCTGATA	
cgl0833-qF	ACATCCACGAGTTCAAGT	
cgl0833-qR	TCATACCGCCAAGAAGAA	

Supplementary references

1. Zhang, Y. et al. A new genome-scale metabolic model of *Corynebacterium glutamicum* and its application. *Biotechnol. Biofuels* **10,** 169 (2017).
2. Tuyishime, P. et al. Engineering *Corynebacterium glutamicum* for methanol-dependent growth and glutamate production. *Metab. Eng.* **49,** 220–231 (2018).
3. Schäfer, A. et al. Small mobilizable multi-purpose cloning vectors derived from the *Escherichia coli* plasmids pK18 and pK19: selection of defined deletions in the chromosome of *Corynebacterium glutamicum*. *Gene* **145,** 69–73 (1994).
4. Kirchner, O. & Tauch, A. Tools for genetic engineering in the amino acid-producing bacterium *Corynebacterium glutamicum*. *J. Biotechnol.* **104,** 287–299 (2003).
5. Wang, Y. et al. MACBETH: multiplex automated *Corynebacterium glutamicum* base editing method. *Metab. Eng.* **47,** 200–210 (2018).
6. Wang, Y. et al. Expanding targeting scope, editing window, and base transition capability of base editing in *Corynebacterium glutamicum*. *Biotechnol. Bioeng.* **116,** 3016–3029 (2019).
