# Supplementary material for: Adaptive laboratory evolution enhances methanol tolerance and conversion in engineered Corynebacterium glutamicum
Source: Commun Biol. 2020 May 7;3:217. doi: 10.1038/s42003-020-0954-9 (PMC7205612; doi:10.1038/s42003-020-0954-9)
Supplement: Supplementary file 3 — Description of Additional Supplementary Files [file 42003_2020_954_MOESM3_ESM.pdf]

Description of Additional Supplementary Files

File Name: **Supplementary Data 1**

Description: Gene transcript level changes between C. glutamicum strain MX14 cultivated with 15 g/L vs. 4 g/L methanol Supplementary Data 1 is provided separately as an excel document
