# Supplementary material for: Adaptive laboratory evolution enhances methanol tolerance and conversion in engineered Corynebacterium glutamicum
Source: Commun Biol. 2020 May 7;3:217. doi: 10.1038/s42003-020-0954-9 (PMC7205612; doi:10.1038/s42003-020-0954-9)
Supplement: Supplementary file 5 — Reporting Summary [file 42003_2020_954_MOESM5_ESM.pdf]

nature research

Corresponding author(s): Ping Zheng

Last updated by author(s): Mar 25, 2020

Reporting Summary

Nature Research wishes to improve the reproducibility of the work that we publish. This form provides structure for consistency and transparency in reporting. For further information on Nature Research policies, see Authors & Referees and the Editorial Policy Checklist.

Statistics

For all statistical analyses, confirm that the following items are present in the figure legend, table legend, main text, or Methods section.

n/a	Confirmed	
☐	☒	The exact sample size (*n*) for each experimental group/condition, given as a discrete number and unit of measurement
☐	☒	A statement on whether measurements were taken from distinct samples or whether the same sample was measured repeatedly
☐	☒	The statistical test(s) used AND whether they are one- or two-sided<br>*Only common tests should be described solely by name; describe more complex techniques in the Methods section.*
☒	☐	A description of all covariates tested
☒	☐	A description of any assumptions or corrections, such as tests of normality and adjustment for multiple comparisons
☐	☒	A full description of the statistical parameters including central tendency (e.g. means) or other basic estimates (e.g. regression coefficient) AND variation (e.g. standard deviation) or associated estimates of uncertainty (e.g. confidence intervals)
☐	☒	For null hypothesis testing, the test statistic (e.g. *F*, *t*, *r*) with confidence intervals, effect sizes, degrees of freedom and *P* value noted<br>*Give P values as exact values whenever suitable.*
☒	☐	For Bayesian analysis, information on the choice of priors and Markov chain Monte Carlo settings
☒	☐	For hierarchical and complex designs, identification of the appropriate level for tests and full reporting of outcomes
☒	☐	Estimates of effect sizes (e.g. Cohen's *d*, Pearson's *r*), indicating how they were calculated

Our web collection on statistics for biologists contains articles on many of the points above.

Software and code

Policy information about availability of computer code

Data collection	Illumina Hiseq Control software was used on the Illumina Hiseq sequencers to collect the sequencing data
Data analysis	FASTQC software (v.0.10.1) was used to assess the quality of raw sequence reads. Based on the quality assessment results, low-quality reads and bases from both ends of raw Illumina reads were removed and trimmed using the NGSQC Toolkit (v.2.3.3) (-l 70, -s 25). After the high-quality reads were aligned against the C. glutamicum ATCC 13032 reference genome (GenBank accession number GCA_000011325.1) using BWA alignment software (v.0.7.17), the mapping results were sorted and indexed using SAM tools software (v.1.9). Raw read counts from BAM files were obtained using HTSeq software (v.0.11.2). The raw-count table was further processed with the DESeq function of the DeSeq2 package (v.1.18.1) to obtain gene expression data. Genes with an adjusted P-value<0.05 and log2(Fold change)>0.5 or <-0.5 were considered to be differentially expressed. Pearson's linear correlation coefficients between variables was calculated using the R package 'stats' and plotted using 'corrplot'. Principal component analysis was performed in 'stats' package and plotted using 'ggord' package.

For manuscripts utilizing custom algorithms or software that are central to the research but not yet described in published literature, software must be made available to editors/reviewers. We strongly encourage code deposition in a community repository (e.g. GitHub). See the Nature Research guidelines for submitting code & software for further information.

Data

Policy information about availability of data

All manuscripts must include a data availability statement. This statement should provide the following information, where applicable:

- Accession codes, unique identifiers, or web links for publicly available datasets
- A list of figures that have associated raw data
- A description of any restrictions on data availability

The sequencing data were deposited into the Sequence Read Archive (SRA) database (accession number: PRJNA615290) at the National Center for Biotechnology Information (NCBI). All the other data and materials that support the findings of this study are available from the corresponding author on reasonable request.

Field-specific reporting

Please select the one below that is the best fit for your research. If you are not sure, read the appropriate sections before making your selection.

- [x] Life sciences
- [ ] Behavioural & social sciences
- [ ] Ecological, evolutionary & environmental sciences

For a reference copy of the document with all sections, see nature.com/documents/nr-reporting-summary-flat.pdf

Life sciences study design

All studies must disclose on these points even when the disclosure is negative.

Sample size	Sample sizes were determined based on literature precedence for metabolic engineering experiments.
Data exclusions	No data were excluded from the analyses.
Replication	All experiments were repeated at least once. All attempts at replication were successful.
Randomization	E. coli and C. glutamicum strains used in this study were grown under identical conditions; no randomization was used.
Blinding	E. coli and C. glutamicum strains used in this study were grown under identical conditions; blinding was not used.

Reporting for specific materials, systems and methods

We require information from authors about some types of materials, experimental systems and methods used in many studies. Here, indicate whether each material, system or method listed is relevant to your study. If you are not sure if a list item applies to your research, read the appropriate section before selecting a response.

Materials & experimental systems

n/a	Involved in the study	
☒	☐	Antibodies
☒	☐	Eukaryotic cell lines
☒	☐	Palaeontology
☒	☐	Animals and other organisms
☒	☐	Human research participants
☒	☐	Clinical data

Methods

n/a	Involved in the study	
☒	☐	ChIP-seq
☒	☐	Flow cytometry
☒	☐	MRI-based neuroimaging
